# Driving forces for COVID-19 clinical trials using chloroquine: the need to choose the right research questions and outcomes

**DOI:** 10.1590/0037-8682-0155-2020

**Published:** 2020-04-06

**Authors:** Wuelton Marcelo Monteiro, Jose Diego Brito-Sousa, Djane Baía-da-Silva, Gisely Cardoso de Melo, André Machado Siqueira, Fernando Val, Cláudio Tadeu Daniel-Ribeiro, Marcus Vinicius Guimarães Lacerda

**Affiliations:** 1 Fundação de Medicina Tropical Dr. Heitor Vieira Dourado, Departamento de Ensino e Pesquisa, Manaus, AM, Brasil.; 2 Universidade do Estado do Amazonas, Programa de Pós-Graduação em Medicina Tropical, Manaus, AM, Brasil.; 3 Instituto Nacional de Infectologia Carlos Chagas, Rio de Janeiro, RJ, Brasil.; 4 Fundação Oswaldo Cruz, Instituto Oswaldo Cruz, Rio de Janeiro, RJ. Brasil.; 5 Fundação Oswaldo Cruz, Instituto de Pesquisas Leônidas e Maria Deane, Manaus, AM, Brasil.

Dear Editor:

The first cases of the new coronavirus (COVID-19) were reported in December, 2019, when a group of patients was admitted to hospitals with an initial diagnosis of pneumonia of unknown etiology^1^. Initially, the outbreak of the new SARSCoV-2 coronavirus (coronavirus disease 2019; formerly 2019-nCoV) was centralized in the province of Hubei, Republic of China, and later spread to many other countries^2^. SARS-CoV-2 infection appears to cause a wide range of symptoms, encompassing asymptomatic infection, mild infections of the upper respiratory tract, severe viral pneumonia, respiratory failure, multiple organ failure and death^3^. Some studies have shown detailed clinical features of some patients with SARS-CoV-2^4^. Of the 44,672 laboratory confirmed patients in China, almost 5% had critical illnesses and almost 50% of the critical patients died, with the overall rate of fatal cases (2.3%) being higher than that observed for seasonal influenza^5^. Most deaths involved older adults, many of whom had underlying chronic diseases^4^ ^,^ ^6^, while children are less likely to develop severe infections^7^. Despite there being no available data so far, anecdotal data from Italy showed a huge number of deaths in the elderly, paving the way for drastic control measures worldwide and compassionate use of drugs in severe cases. 

Chloroquine (CQ) was unequivocally demonstrated to have *in vitro* inhibiting effects on SARS-CoV-2 infection^8^ and was precociously publicized as having a beneficial effect in COVID-19 patients after a study conducted in Marseille, France showed a viral load reduction in COVID-19 patients^9^. Since there is no specific antiviral therapy for coronavirus infections to date, the announcement of partial and fragile data led to precipitated political manifestations by major government leaders and contributed to uncoordinated recommendations of the drug to severe patients. Considering CQ’s low costs, good safety profile, *in vitro* activity against other viral diseases, preexisting supply chain with potential for public and private augmented production, and knowledge on specificity and management of side effects accumulated for decades of use for anti-malarial use, it is expected that clinical assays will be promptly designed to multicentrically evaluate the actual potential of the drug for treating severe COVID-19 cases.

CQ sulfate and phosphate salts and hydroxychloroquine (HCQ) have both been used as antimalarial drugs for decades. HCQ, a derivative of CQ, was first synthesized in 1946 by the introduction of an N-hydroxy-ethyl side chain in place of QC's N-diethyl group and proved to be less (~40%) toxic during prolonged use and is therefore recommended for the treatment of autoimmune diseases, such as systemic lupus erythematosus and rheumatoid arthritis, in which patients are exposed to the drug for months or eventually for years^10^. 

Several studies are currently being carried out with CQ and HCQ, however only a few of the researchers published preliminary results, while other studies were canceled, mainly due to the reduction of cases in China. As of the preparation of this manuscript (March 26^th^, 2020), only 13 trials have been registered on the ClinicalTrials.gov platform, with 2 studies being carried out in Brazil using CQ or HCQ and azithromycin.Additionally, 22 trials have been registered on the Chinese Clinical Trial Registry, of which 6 are stated as having been withdrawn (http://www.chictr.org.cn/index.aspx). The large number of studies conducted with this drug shows that the scientific community is making a great effort to clarify the role of these drugs in reducing the mortality associated with COVID-19, but that effort is probably not being sufficiently well coordinated yet.

Different treatments and lengths of treatment, and dose regimes have been reported. However, there is an urgent need for adaptive clinical trial designs in order to properly - and rapidly - respond to different questions observed during Covid-19 pandemic. Adaptive clinical trials have contributed greatly to advances in patient care byincreasing the efficiency and flexibility of randomized clinical trials, reducing costs and increasing the likelihood of finding a true benefit, if any, of the intervention being studied^11^. Several deficiencies in traditional randomized clinical trials (RCTs) have been observed, including larger sample sizes and lengthy duration, lack of power to evaluate global efficacy or in important subgroups, and cost, which all limit medical innovation especially in our emergency scenario^12^. 

Finally, there is a need for urgent answers that should not be limited to our current scenario, but should also be used to prepare for future Covid-19 pandemics, considering that cases will only be available now and no studies will be feasible in the meantime. This has happened in China, where many trials were interrupted because no more cases were appearing by the time studies were ready to get started. Currently, several obstacles still need to be discussed and addressed (Table 1): the use of a placebo group in severe patients to effectively show treatment efficacy; lack of instantaneous funding; need for timely protocol approval by ethical boards worldwide; the use of multicentric studies rather than single-center studies, and compliancy with good clinical practices, in order to promptly find answers while the pandemic is still ongoing. The use of a placebo in patients with critical disaease, which would be the best way to prove the efficacy of the different interventions being tested, could be considered unethical by many since there are compassionate clinical conducts to treat such cases arund the globe. At the moment, we all believe that such placebo-controlled trials should only be carried out with non-severe cohorts.

**TABLE 1:** Open questions to be answered regarding the use of chloroquine against Covid-19.

Research question	Expected outcome
**Does it work to prevent infection?**	Decrease in confirmed infections in a cohort of uninfected individuals
*Pre- and post-exposure prophylaxis*	
**Does it work to prevent clinical disease?**	Decrease in proportion of patients that develop clinical symptoms
*Clinical disease prevention*	
**Does it work to prevent clinical severity?**	Decrease in proportion of patients with onset of severe disease (SARS criteria, need for respiratory support, ICU admission, post-infection respiratory sequelae)
*Severe clinical disease prevention*	
**Does it work to prevent death?**	Reduced mortality in severe cases (hospitalized patients)
*Mortality prevention*	
**Does it decrease viral load in blood and secretions?**	Rapid viral clearance after treatment
*Viral load*	
